# Generation and Applications of a DNA Aptamer against Gremlin-1

**DOI:** 10.3390/molecules22050706

**Published:** 2017-04-28

**Authors:** Qian Li, Yongwei Huo, Yonghong Guo, Xiaoyan Zheng, Wengang Sun, Zhiming Hao

**Affiliations:** 1 Department of Rheumatology, the First Affiliated Hospital of Xi’an Jiaotong University, Xi’an 710061, China; liqianlbt@126.com (Q.L.); xiaoy_2008@126.com (X.Z.); 15829307167@139.com (W.S.); 2 Research Center of Reproductive Medicine, Medical School of Xi’an Jiaotong University, Xi’an 710061, China; hyw301@mail.xjtu.edu.cn; 3 Department of Infectious Diseases, the Second Affiliated Hospital of Xi’an Jiaotong University, Xi’an 710061, China; xiaoqing9759@sina.com

**Keywords:** aptamer, Gremlin-1, South-Western blot analysis, enzyme-linked aptamer sorbent assay, immunohisto-cytochemical staining

## Abstract

Gremlin-1, a highly conserved glycosylated and phosphorylated secretory protein, plays important roles in diverse biological processes including early embryonic development, fibrosis, tumorigenesis, and renal pathophysiology. Aptamers, which are RNA or DNA single-stranded oligonucleotides capable of binding specifically to different targets ranging from small organics to whole cells, have potential applications in targeted imaging, diagnosis and therapy. In this study, we obtained a DNA aptamer against Gremlin-1 (G-ap49) using in vitro Systematic Evolution of Ligands by Exponential Enrichment (SELEX). Binding assay and dot-blot showed that G-ap49 had high affinity for Gremlin-1. Further experiments indicated that G-ap49 was quite stable in a cell culture system and could be used in South-Western blot analysis, enzyme-linked aptamer sorbent assay (ELASA), and aptamer-based cytochemistry and histochemistry staining to detect Gremlin-1. Moreover, our study demonstrated that G-ap49 is capable of revealing the subcellular localization of Gremlin-1. These data indicate that G-ap49 can be used as an alternative to antibodies in detecting Gremlin-1.

## 1. Introduction

Gremlin-1 (or down-regulated by mos, Drm), a highly conserved cysteine knot-secreted glycoprotein, contains 184 amino acids and belongs to the differential screening-selected gene aberrant in neuroblastoma (DAN) family [1,2]. As an antagonist of bone morphogenetic proteins (BMPs), Gremlin-1 binds to BMP-2, -4, and -7 and inhibits their activities [3,4,5,6,7]. Gremlin-1 also binds to vascular endothelial growth factor receptor-2 (VEGFR-2) [8], leading to VEGFR-2-dependent angiogenesis [9]; interacts with tyrosine 3-monooxygenase/tryptophan 5-monooxygenase activation protein eta (YWHAH), playing a role in human tumorigenesis [10]; and combines to Slit proteins (a family of secreted extracellular matrixproteins which play an important signaling role in the neural development of most bilaterians) [7], strongly potentiating the inhibitory activity on leucocyte chemotaxis [1,7]. During embryonic development, highly expressed Gremlin-1 regulates diverse biological processes such as limb and metanephric kidney organogenesis [3]. Abnormally high expression of Gremlin-1 is closely correlated with idiopathic pulmonary fibrosis (IPF) [11,12] and human renal diseases such as diabetic nephropathy (DN) [13], pauci-immune glomerulonephritis [14,15] and chronic allograft nephropathy [15]. Moreover, Gremlin-1 is involved in the development of sarcoma and carcinomas of the breast, uterine cervix, ovary, lung, pancreas, kidney and colon [8,16].

Aptamers are short single-stranded RNA or DNA molecules capable of tight and specific binding to their targets due to the formation of characteristic spatial structures. In 1990, three independent laboratories described the isolation of RNA aptamers by Systematic Evolution of Ligands by Exponential enrichment (SELEX) technology [17,18,19]. Afterwards, RNA and DNA aptamers have been explored extensively as specific and high affinity probes to a variety of targets, ranging from small organic molecules to large biomolecules like proteins and even cells [20,21,22]. Similar to antibodies, aptamers can strongly interact with their targets and may affect the biological functions of targets [20,21,22]. In addition, aptamers offer many potentially significant advantages over antibodies, such as no immunogenicity, rapid tissue penetration, thermal stability, low cost, convenient synthesis and easy modification [20]. Thus, a number of aptamers have been explored for biomarker discovery, diagnosis, precisely controlled drug release and targeted therapy [20,21,22]. The anti-VEGF165 RNA aptamer, Petapganib, is the first to be successfully used in the therapy of ocular vascular diseases [23]. Moreover, more than a dozen of other aptamers are being evaluated in clinical trials [22].

In this study, we generated a DNA aptamer of high affinity against Gremlin-1 (G-ap49) using SELEX iterative method. Further tests verified that G-ap49 can be used in South-Western blot, immunohisto(cyto)chemistry and enzyme-linked aptamer sorbent assay (ELASA) for the detection of Gremlin-1. Moreover, our study demonstrated that G-ap49 can be used in revealing the subcellular localization of Gremlin-1 as well as the anti-Gremlin-1 antibody.

## 2. Results

### 2.1. Selection and Characterization of G-ap49 Specific to Gremlin-1

After 15 rounds of selection, the bound ssDNA was eluted, which was subsequently used as a template to yield dsDNA. The dsDNA pool was cloned into pGEM-T easy vector for isolating single sequences. We picked 50 colonies for binding assay, to further obtain aptamer candidates with higher affinity. The top 10 candidates were chosen for sequence analysis. The results showed that among these 10 colonies, four colonies (G-ap32, G-ap39, G-ap48, G-ap49) had similar sequences while the others six candidates had diverse sequences. Among the former four candidates, G-ap49 had the lowest free energy according to the secondary structure prediction, thus we used G-ap49 for further identification and application experiments.

**Figure 1:**
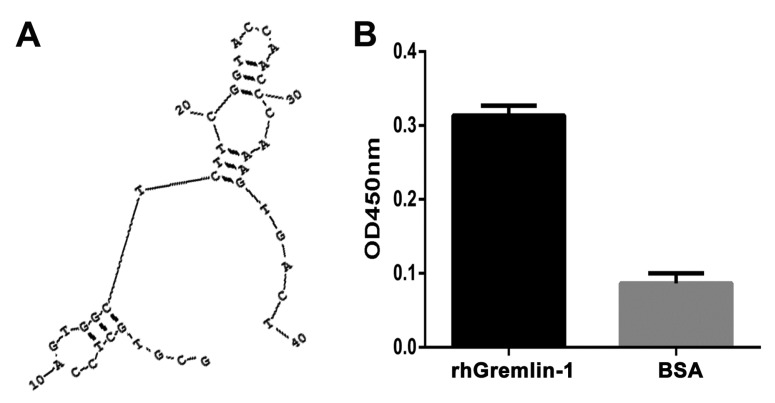

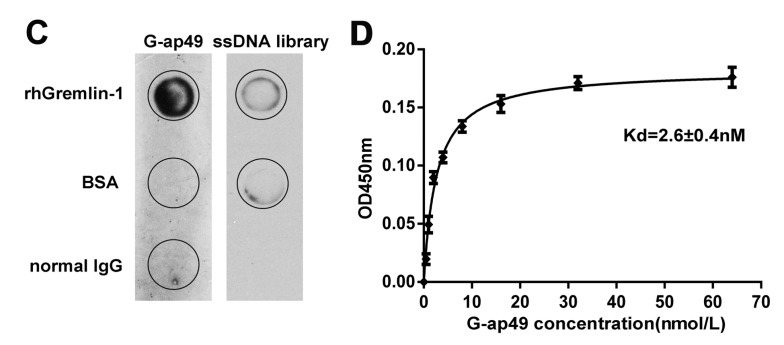
Characterization of G-ap49, an anti-Gremlin-1 DNA aptamer. (**A**) RNA Structure v3.5 prediction showed that G-ap49 folded into a two-stem-loop secondary structure; (**B**) Binding assay displayed that G-ap49 had high affinity to Gremlin-1; (**C**) Dot-blot verified that G-ap49 specifically bound to membrane-immobilized Gremlin-1; (**D**) G-ap49 had a low equilibrium dissociation constants (Kd) of 2.6 ± 0.4 nM.

As shown in the predicted secondary structure (Figure 1A), G-ap49 without flanking sequences (GCGTGCTCCAGTGGCTCTTCGGTACCAACCCAAAGTGACT) folded into a stable structure of two stem-loops linked by a T. Binding assay and dot-blot verified that G-ap49 specifically bound to Gremlin-1 (Figure 1B,C). Equilibrium dissociation constants (Kd) of G-ap49 were determined by increasing G-ap49 concentrations with a fixed Gremlin-1 concentration. The Kd value of G-ap49 was 2.6 ± 0.4 nM, clustering in low-nanomolar range, which indicated the high affinity to Gremlin-1 (Figure 1D).

### 2.2. Both G-ap49 and Anti-Gremlin-1 Antibody Are Effective to Detect Gremlin-1

**Figure 2:**
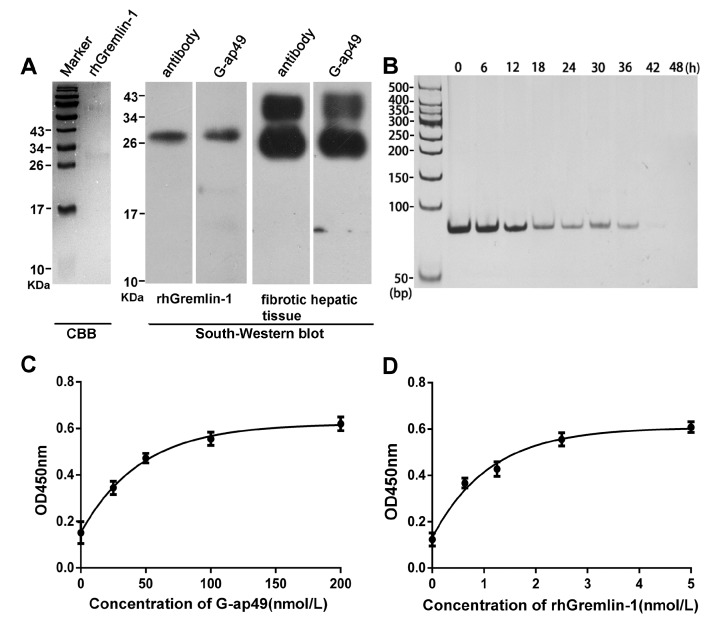
Applications of G-ap49 in South-Western blot and enzyme-linked aptamer sorbent assay (ELASA). (**A**) South-Western blot showed that G-ap49 recognized membrane-bound Gremlin-1 (200 ng), just as a specific anti-Gremlin-1 antibody while in SDS-PAGE followed by Coomassie Brilliant Blue (CBB) R250 staining, the rhGremlin-1 band was barely visible; (**B**) G-ap49 was quite stable under cell culture system; (**C**) Sandwich ELASA was used to determine a proper G-ap49 concentration; (**D**) ELASA with different Gremlin-1 concentrations and a constant amount of biotinylated G-ap49 (100 nM).

To show whether G-ap49 could be used as a specific and sensitive probe for detection, we performed South-Western blot to explore whether G-ap49 could be used to detect denatured, membrane-bound Gremlin-1. The initial result with rhGremlin-1 demonstrated that G-ap49 recognized a 25 kDa band, which was identical to the band probed by a Gremlin-1 specific antibody, after SDS-PAGE and membrane transfer. Furthermore, South-Western blot with fibrotic hepatic tissues verified that G-ap49 recognized a band of 25 kDa corresponding to that of rhGremlin-1, and a band of larger molecular weight (about 31 kDa). Western blot with the same tissue using Gremlin-1 specific antibody also showed an identical result (Figure 2A). These results verified that South-Western blot with G-ap49 could be used to detect tissue Gremlin-1 in replacement of Western blot with a specific antibody.

A sandwich ELASA assay was established to measure Gremlin-1 concentration in liquids, mimicking sandwich enzyme-linked immunosorbent assay (sandwich ELISA). The system used anti-Gremlin-1 rabbit polyclonal antibody as the capture antibody for Gremlin-1, biotinylated G-ap49 and HRP-conjugated streptavidin as the detection system. The test began with assessing the stability of G-ap49 in complete cell culture medium. As shown in Figure 2B, although the concentration of G-ap49 decreased over time, it still could be detected at 36 h after addition, indicating that the aptamer was quite stable in a cell culture system. Then, with a fixed Gremlin-1 concentration, we titrated the proper concentration of G-ap49 in this system (Figure 2C). Based on the results, 100 nM was determined as the working concentration of G-ap49. Under this condition, a nice linear relationship between the concentration of Gremlin-1 and the optical density was observed (Figure 2D), indicating that G-ap49 could serve as the second antibody in sandwich ELASA technique.

### 2.3. G-ap49 Was Optimal to Detect Gremlin-1 in Immunohisto-Cytochemical Staining

**Figure 3:**
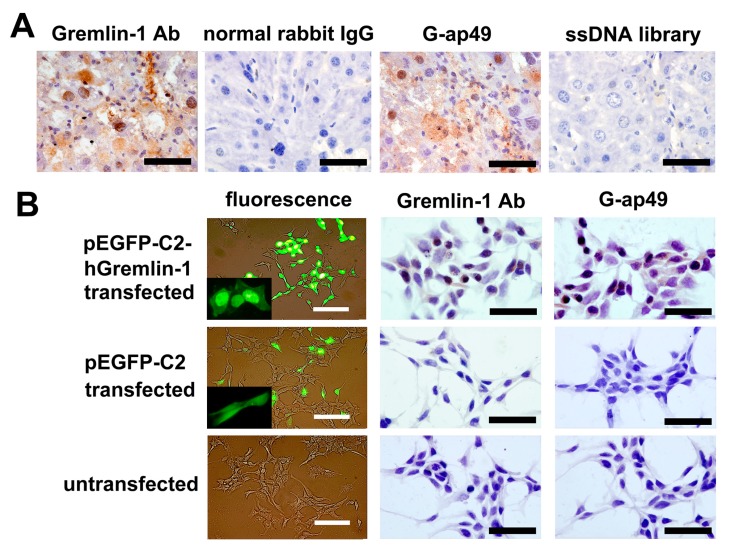
Application of G-ap49 in cytochemistry and histochemistry staining. (**A**) Biotinylated G-ap49 was used instead of the primary antibody to probe the expression of Gremlin-1 in CCl_4_-induced fibrotic mouse hepatic tissue. Rabbit anti-Gremlin-1 polyclonal antibody served as a positive control. Normal rabbit IgG and biotinylated library ssDNA served as negative controls; (**B**) G-ap49-based cytochemistry confirmed the specificity of G-ap49 in in situ detection of the expression of Gremlin-1. G-ap49 positive staining only existed in pEGFP-C2-hGremlin-1-transfected HEK293T cells, which was consistent with the fluorescence observation and immunostaining with anti-Gremlin-1 antibody. Scale bars = 50 μm.

To further investigate the potential applications of G-ap49 versus anti-Gremlin-1 specific antibody, we evaluated the use of G-ap49 as a probe in formalin-fixed, paraffin-embedded tissues. In mouse fibrotic hepatic tissue, G-ap49 and anti-Gremlin-1 antibody exhibited similar positive staining in fibrous septa and portal areas. Interestingly, a few of hepatocytes showed positive nuclear staining with G-ap49 (Figure 3A) as well as anti-Gremlin-1 antibody. To further confirm this result, HEK293T cells, which have no or low-level expression of Gremlin-1, were transfected with a plasmid carrying EGFP-hGremlin-1 fusion gene. Fluorescence observation, G-ap49-based cytochemical staining, and immunocytochemistry with anti-Gremlin-1 antibody showed high consistence (Figure 3B). These results clearly verify that G-ap49 may advantageously replace anti-Gremlin-1 specific antibody in immunohisto-cytochemical staining.

## 3. Discussion

Gremlin-1 is a multifunctional protein which is critical in embryonic development, homeostasis maintaining and a number of diseases. Altered Gremlin-1 levels in tissues [11,13,24], blood [25] and body fluids [26] have been reported in various pathological conditions, such as malignancies [16], fibrosis [12,24] and inflammatory disorders [27]. Thus, finding a proper method to determine the concentration of Gremlin-1 is important in the diagnosis of these diseases.

Currently, the determination of Gremlin-1 at protein level solely depends on its antibodies. Nucleic acid aptamers emerged as new targeting probes with excellent potential in targeted imaging, diagnosis and therapy. This anti-Gremlin-1 DNA aptamer, G-ap49, which showed a high affinity (2.6 ± 0.4 nM) and good specificity for Gremlin-1, provides an alternative tool for the determination of Gremlin-1 using South-Western blot analysis, ELASA, and aptamer-based cytochemistry and histochemistry staining. Besides, G-ap49 can provide a non-expensive and easy-to-manufacture ELASA kit for the determination of Gremlin-1 in body fluids.

Although antibodies were considered as the most specific probes for targets detection due to their high affinity and their good specificity, the aptamer against Gremlin-1 we generated still has some merits. In addition to its thermal stability, low cost, convenient synthesis and easy to modify, G-ap49 can penetrate into subcellular organelles easily due to its small molecular weight. Thus, G-ap49-based cytochemistry and histochemistry might provide accurate subcellular localization of Gremlin-1. In the present study, both G-ap49-based histochemistry and immunohistochemistry with anti-Gremlin-1 antibody showed that a few hepatocytes were positively stained. Our subsequent experiments demonstrated that Gremlin-1 possessed of a bipartite nuclear localization signal which could direct Gremlin-1 to the nucleus (our unpublished data), confirming the accuracy of G-ap49-based histochemistry. These results demonstrate that G-ap49 can be used in revealing the subcellular localization of Gremlin-1. However, stringent standardization of the reagents and procedure is required.

## 4. Materials and Methods

### 4.1. Initial Library and Primers for SELEX

The single-stranded DNA (ssDNA) library, PCR primers and biotin-conjugated aptamer candidates were synthesized by Takara, Dalian, China. The ssDNA library contained a central region with a 40-nucleotide (nt) random sequence, flanked by the 18-nucleotide constant region at 5′- and 3′-ends, respectively (5′-GGACAAGAATCACCGCTC-N40-CGTACAGGAGGCATACAG-3′). The primers for the amplification of double-stranded DNA and the final cloning were 5′-GGACAAGAATCACCGCTC-3′ (forward) and 5′-CTGTATGCCTCCTGTACG-3′ (reverse). 5′-end biotinylated forward primer was also prepared for the initial identification of the affinities of the aptamer candidates.

### 4.2. SELEX Procedure

96-well polystyrene microplates (Thermo Scientific, Roskilde, Denmark) were coated with recombinant human Gremlin-1 (rhGremlin-1, R&D Systems, Minneapolis, MN, USA) at 50 ng/well diluted in 50 mM carbonate buffer, pH 9.6, at 4 °C overnight. They were then blocked with 3% bovine serum albumin (BSA) dissolved in SHMCK buffer (20 mM Hepes, 120 mM NaCl, 5 mM KCl, 1 mM MgCl_2_ and 1 mM CaCl_2_, pH 7.4) at 37 °C for 2 h. The ssDNA library was dissolved in SHMCK buffer (2 μg/mL), denatured at 95 °C for 10 min, immediately cooled on ice for 10 min, and renatured at room temperature for 10 min. Then yeast tRNA and BSA (both from Sigma-Aldrich, St. Louis, MO, USA) were added to the renatured ssDNA library to reach final concentrations of 100 μg/mL and 1 mg/mL, respectively. Upon selection, 100 μL of the ssDNA mixture was added into a 3% BSA-coated well and incubated at 37 °C for 1 h. Next, the supernatant was shifted to a Gremlin-1-coated well and maintained at 37 °C for another 1 h. Unbound ssDNA were removed by washing three times with SHMCK buffer supplemented with 0.05% Tween-20 (SHMCKT). Finally, the bound ssDNA was eluted with 100 μL of elution buffer (7 M urea, 0.5 M NH_4_Ac, 1 mM EDTA, 0.2% SDS) at 95 °C for 10 min. After phenol-chloroform-isoamyl alcohol (25:24:1) extraction, the precipitate was dissolved in TE buffer (10 mM Tris-HCl, 1 mM EDTA, pH 8.0).

The dissociated ssDNA was amplified by PCR (5 min at 94 °C, followed by 15 cycles of denaturation at 94 °C for 30 s, anneal at 59 °C for 30 s and extension at 72 °C for 30 s). Agarose gel-purified PCR product (double-stranded DNA, dsDNA) was used as the template in another 15-cycle asymmetric PCR (only forward primer was added) to generate ssDNA. After separation by 10% denatured polyacrylamide gel electrophoresis (PAGE) and extraction using EZ Spin Column PAGE Oligo Gel DNA Extraction Kit (Sangon Biotech, Shanghai, China), purified ssDNA were quantified using an OD260/280 ratio (Bio-Rad, Hercules, CA, USA), which served as the template in the next round of selection.

### 4.3. Cloning, Aptamer Binding Assay, DNA Sequencing and Secondary Structure Prediction

After 15 rounds of selection, the purified dsDNA were cloned into pGEM T-Easy (Promega, Madison, WI, USA) and transformed into *E. coli* (DH5α). Fifty colonies were picked for the extraction of the recombinant plasmids.

Biotinylated aptamer candidates with flanking sequences were produced by asymmetric PCR using the 5′-end biotin-labeled forward primer. The recombinant pGEM-T plasmids served as the template. The reconstitution of the biotinylated aptamer candidates and binding test were performed just as in the case of selection. After three washes with SHMCKT, horseradish peroxidase (HRP)-conjugated streptavidin (1:2000 diluted in SHMCK, Cell Signaling Technology, Danvers, MA, USA) was added to the wells and the reaction was maintained at 37 °C for 1 h. Followed by three washes with SHMCKT, 3,3′,5,5′-Tetramethylbenzidine (TMB) substrate complex (Sigma-Aldrich, St. Louis, MO, USA) was added and the absorbance at 450 nm was measured. Binding experiments were performed in triplicate. Finally, the top 10 recombinant plasmids containing aptamer candidates with high affinities were selected for sequence analysis. The secondary structures of the sequences were predicted by using RNA Structure v3.5 (Mathews Lab, Rochester, NY, USA).

### 4.4. Dot-Blot

Dot-blot was employed to confirm the affinity and specificity of the aptamer candidates. Gremlin-1 was dotted at 200 ng per spot on a polyvinylidene fluoride (PVDF) membrane. An equal amount of BSA and normal IgG served as a control. After being blocked with 10% skim milk in phosphate-buffered saline supplemented with 0.05% Tween-20 (PBST), the membrane was incubated with renatured 5′-end biotin-labeled ssDNA without flanking sequences (diluted to 80 nM in PBS) at 37 °C for 1 h. After three washes with PBST, the membrane was incubated with HRP-conjugated streptavidin (1:2000 diluted in PBST), and finally the blots were developed by enhanced chemiluminescence (ECL, Santa Cruz Biotechnology, Santa Cruz, CA, USA).

### 4.5. Determination of Equilibrium Dissociation Constants (Kd)

Equilibrium dissociation constants (Kd) of the selected aptamer was determined by measuring the binding of different concentrations of the 5′-end biotin-labeled aptamer (0 nM, 0.5 nM, 1 nM, 2 nM, 4 nM, 8 nM, 16 nM, 32 nM, 64 nM diluted in SHMCK buffer) with a constant amount of Gremlin-1 (50 ng/well). Kd was calculated by fitting the binding data to a one-site saturation equation; Y = Bmax × X/(Kd + X), in GraphPad Prism software 6.0 (GraphPad Software Inc., La Jolla, CA, USA).

### 4.6. South-Western Blotting

CCl_4_-induced mouse fibrotic hepatic tissues were lysed in radioimmunoprecipitation (RIPA) lysis buffer supplemented with phosphatase and protease inhibitors. A total of 100 μg of the extracted protein or 200 ng of rhGremlin-1 was applied to a 12% SDS-PAGE and transferred onto a PVDF membrane. The membrane was incubated with chemically synthesized, biotin-labeled aptamer (80 nM in PBS) at 4 °C overnight, followed by rinsing three times with PBST. The bound aptamer was then recognized by HRP-streptavidin (1:2000 diluted in PBST) using ECL. Western blot with rabbit anti-Gremlin-1 polyclonal antibody (1:500 diluted in PBST, Sangon Biotech, Shanghai, China) was also performed to confirm the results. To exclude a non-specific binding, a gel with 200 ng rhGremlin-1 was stained with Coomassie brilliant blue R250.

### 4.7. ELASA

We tried to develop a sandwich ELASA method to determine Gremlin-1 with the aptamer and a Gremlin-1 antibody. Firstly, we determined a proper concentration of the biotin-labeled aptamer to be used in this assay. Polystyrene microplates were coated with anti-Gremlin-1 rabbit polyclonal antibody at 4 °C overnight and blocked with 3% BSA in PBST. rhGremlin-1 was added at a concentration of 2.5 nmol/L and incubated at 37 °C for 1 h, followed by three PBST washes. Then, the wells were incubated with the aptamer at different concentrations (200 nM, 100 nM, 50 nM, 25 nM, 0 nM diluted in SHMCK buffer) at 37 °C for 1 h, washed in PBST, and incubated with horseradish peroxidase (HRP)-conjugated streptavidin at 37 °C for 1 h. Finally, TMB substrate complex was added and the absorbance at 450 nm was measured.

Based on the results of the above test, we tried sandwich ELASA to measure the concentration of Gremlin-1 in fluids. The test was carried out as described above except that different Gremlin-1 concentrations (5 nM, 2.5 nM, 1.25 nM, 0.63 nM, 0 nM diluted in 3% BSA/PBST) and a constant amount of the biotinylated aptamer (100 nM) were used. Three independent experiments were performed in triplicate.

### 4.8. Aptamer-Based Histochemistry and Cytochemistry

For aptamer-based histochemical staining, the formalin-fixed and paraffin-embedded mouse fibrotic hepatic tissue (CCl_4_-induced) sections were deparaffinized, rehydrated routinely and then rinsed in distilled water. The sections were boiled in 10 mM sodium citrate buffer (pH 6.0) at 95 °C for 10 min for antigen retrieval, followed by incubation in 3% H_2_O_2_ at room temperature for 30 min to quench the endogenous peroxidase. Then biotinylated G-ap49 (400 nM final concentration in PBS) was added and the reaction was maintained overnight at 4 °C. After three washes with PBST, the tissue sections were incubated with HRP-conjugated streptavidin (1:500 diluted in PBST) at 37 °C for 1 h. After another three washes, the positive staining was visualized with DAB followed by counterstaining with hematoxylin. At the same time, the biotinylated ssDNA library served as a negative control. To confirm the aptamer-based histochemical staining, immunohistochemistry with rabbit anti-Gremlin-1 polyclonal Ab (1:200 diluted in PBST) using Histostain™-Plus SP kit (Beijing Zhongshan Goldenbridge Company, Beijing, China) was also performed, in which normal rabbit IgG were used as a negative control.

HEK293T cells with ectopic expression of hGremlin-1 were used to test the usability of the aptamer in cytochemical staining. One day before transfection, cells were seeded into a six-well plate with sterile coverslips placed on the bottom of the wells. Transfection of recombinant plasmids pEGFP-C2-hGremlin-1, which contained EGFP-hGremlin-1 fusion gene in frame, and pEGFP-C2 were carried out using Lipofectamine 2000 (Invitrogen, Carlsbad, CA, USA) following the manufacturer’s instructions. Two days later, transfection efficiency was verified by observing the expression of EGFP under a fluorescence microscope (Olympus, Tokyo, Japan). Then, the coverslips were fixed with 4% paraformaldehyde, permeabilized with 0.5% Triton X-100, and stored at −20 °C until use. The coverslips were treated in the same manner as those used for histochemical staining.

### 4.9. Biological Stability Assay

To test the stability of G-ap49 in biological fluids, 1 μg of G-ap49 with flanking sequences was added into a well of a 96-well plate in which HEK293T cells were cultured with 200 μL Dulbecco’s modified Eagle’s medium (DMEM, Gibco, Grand Island, NY, USA) supplemented with 10% fetal bovine serum (FBS, Gibco). Five-microliter culture media was sampled at 6-h intervals. After PCR with the sample serving as a template, PCR products were analyzed by PAGE-silver staining to evaluate the remaining levels of G-ap49.

## 5. Conclusions

We generated a DNA aptamer (G-ap49) with high affinity to Gremlin-1 using SELEX, and our results preliminarily verified that G-ap49 could be used as an alternative to antibodies in the detection of Gremlin-1 in South-Western blot and ELASA assay. Moreover, G-ap49 could penetrated deep into cells as well as the antibody in revealing the subcellular localization of Gremlin-1 inimmunohisto-cytochemical staining. Thus, G-ap49 may advantageously replace the use of a specific antibody in detecting Gremlin-1, due to the lack of immunogenicity and its capacity to be chemically modified. In addition, further experiments are underway in our laboratory to improve G-ap49-based detection measures.
